# Access to HIV treatment during community-wide flooding: Experiences of people living with HIV and health care workers in Malawi, a mixed methods study

**DOI:** 10.1371/journal.pgph.0005862

**Published:** 2026-02-03

**Authors:** Misheck Mphande, R. Paneno, I. Robson, K. Phiri, M. Cornell, J. J. van Oosterhout, D. P. Eisenman, J. Njala, S. Phiri, K. Dovel

**Affiliations:** 1 Partners in Hope, Lilongwe, Malawi; 2 Division of Infectious Diseases, David Geffen School of Medicine, University of California Los Angeles, Los Angeles, United States of America; 3 CIDER (Centre for Integrated Data and Epidemiological Research), School of Public Health, University of Cape Town, Cape Town, South Africa; 4 Division of General Internal Medicine and Health Services Research, David Geffen School of Medicine, University of California Los Angeles, Los Angeles, United States of America; 5 School of Global and Public Health, Kamuzu University of Health Sciences, Lilongwe, Malawi; London School of Hygiene and Tropical Medicine, UNITED KINGDOM OF GREAT BRITAIN AND NORTHERN IRELAND

## Abstract

Community-wide flooding in high HIV-burdened districts affects continuity of HIV care, especially in resource-limited countries in sub-Saharan Africa. We explored the lived experiences of antiretroviral therapy (ART) clients and health care workers (HCWs) in Malawi to understand challenges and potential solutions for ART delivery during floods. This was a mixed methods study. In quantitative analysis, we used routine program data from Partners in Hope (PIH) to compare interruption in treatment (IIT) (>28 days without medication) cases in months with and without flooding, October 2021 to December 2023, to understand how flooding influences IIT. In qualitative analysis, we conducted in-depth interviews (IDIs) with ART clients and focus group discussions (FGDs) with HCWs from from six health facilities impacted by flooding in Chikwawa, Nsanje and Mulanje districts. We analyzed data in Atlas.Ti. IIT was far higher in flooding periods. Across 49,152 IIT cases, IIT increased by 24% during the first flooding period and by 23% during the second flooding period. 23 ART clients completed IDIs between July and August 2024: 13 female, median age 40 years, predominantly subsistence farmers (n = 21). A total of 9/23 clients experienced IIT. Flooding negatively impacted nearly all IDI participants: 17/23 lost homes and had to relocate, mainly to government camps. Most clients lost crops, livestock, and ART medication. Many clients travelled to find income and despite uncertainties they were motivated to remain in care. In six FGDs with 34 HCWs, HCWs described facility-level ART stock-outs and loss of medical records, limited stakeholder coordination and the absence of guidelines for HIV services during emergencies. HCWs were also impacted but received little support. IIT cases greatly increased during flooding periods, with both clients and HCWs facing multiple challenges to ART service provision. National flooding guidelines, coordination of stakeholder response, adequate planning and seasonal six-month ART dispensing could improve retention.

## Introduction

Extreme weather events like community-wide flooding disproportionately impact vulnerable populations, including people living with HIV (PLHIV) [1,2], causing forced migration and affecting access to health services [3]. Malawi is one of five countries most affected by extreme weather events [4], having experienced three cyclones in the last five years resulting in >500 human deaths and massive damage to the environment and infrastructure [4,5]. The country is ranked the fourth poorest country in the world [6], and is particularly vulnerable to climate change due to extreme poverty, heavy reliance on rain-fed agriculture, and limited flood-resistant infrastructure [7–11]. In settings like Malawi, flooding can hinder provision of HIV care by limiting access to services [10,12–14].

Adult HIV prevalence in Malawi is 8.9%, equating to ~1 million adults living with HIV, 90.4% of whom were on antiretroviral therapy (ART) in 2024 [15–17]. Mitigating the impact of climate change on health care delivery for PLHIV in Malawi is therefore critical. In other contexts, despite severe flooding, PLHIV have been able to sustain ART adherence with adequate health system support [12,18]. However, little is known about the provision of ART services during community-wide flooding in Malawi.

In this paper we describe the experiences of ART clients and healthcare workers (HCWs) in Malawi during flooding and report the interventions they proposed to support sustained ART delivery.

## Methods

### Study design

This study used a sequential mixed methods design [19], first analyzing quantitative routing program data and then analyzing qualitative in-depth interviews (IDIs) with clients and focus group discussions (FGDs) with HCWs.

#### Quantitative methods.

We used quantitative methods to explore the impact of flooding on IIT among clients living with HIV, exploring patterns of IIT before, during and after community-wide flooding events.

### Setting

We identified three districts which were the most affected districts during community-wide flooding: Chikwakwa, Nsanje and Mulanje districts. We further identified six health facilities in these districts that experienced severe flooding, as identified by the Declaration of State of Disaster over Districts affected by Floods and Heavy Rains, January 2022 [20–22]. The facilities were Lengwe and Kakoma in Chikwakwa District, Chambe and Kambenje in Mulanje District, and Mbenje and Ndamera in Nsanje District [22]. These were Malawi Ministry of Health facilities supported by PIH, a Malawian faith-based non-governmental organization (NGO) and PEPFAR/USAID HIV care and treatment implementing partner under the Client-Oriented Response for HIV Epidemic Control (CORE) program. Tropical Storm Ana and Cyclone Gombe, which took place in 2022, were included in the analysis.

### Data collection

We conducted secondary analysis on routine program data collected before, during and after community-wide flooding events between October 2021 to December 2023. Data were aggregated at facility level, providing total counts of ART clients with IIT overtime.

### Data analysis

We defined IIT as missing a scheduled ART clinic appointment for ≥28 days. We compared IIT cases in months with and without flooding over time. We used routinely-collected HIV program data to identify ART clients who did or did not experience IIT and classified them by facility. We conducted descriptive analysis using Microsoft Excel to identify patterns in IIT across flooding episodes.

#### Qualitative methods.

We then used qualitative data to explore experiences of flooding, mechanisms (both barriers and facilitators) that impacted IIT during flooding, and perceived solutions to improve flood responses.

### Data collection

We conducted IDIs with clients and FGDs with HCWs between July and August 2022, seven months after cyclone Gombe. We identified potential IDI participants using purposive sampling, through medical chart reviews. Eligibility criteria included being ≥18 years of age, living in areas impacted by community-wide flooding, and being an ART client. Clients identified as having IIT during flooding (>28 days late for recent ART appointment) (n = 12) were matched with clients who did not experience IIT (n = 11) by gender, age and village.

Identified clients were traced and interviewed in community-based locations and at times suitable for the participants. Interviews were conducted by trained research assistants in the local language (usually Chichewa) and lasted 30–60 minutes. An interview guide was developed to explore clients’ experiences with the health system, challenges, coping mechanisms and recommendations for improving quality of care during flooding.

Focus groups with HCWs were used to triangulate client data and provide a multi-level perspective to lived experiences of flooding and IIT. Using purposive sampling, we conducted FGDs with HCWs (clinical officers and nurses) and Treatment Supporters (TSs), a lay cadre who provide counselling and treatment support and trace clients with IIT. Eligibility criteria included being >18 years of age and working in the ART department of participating facilities during the flooding period. We recruited HCWs at health facilities and conducted FGDs in a private facility room of their choosing. The FGD guide explored HCW challenges, health system challenges and potential solutions to retain clients on ART during flooding. FGDs lasted ~60 minutes and were conducted in the local language (Chichewa) by a trained research assistant.

Participants provided oral consent individually after the consent form was read aloud to them in the local language. This process was audio recorded. In FGDs, study participants were given participant numbers and were referred to using numbers during interviews. This consenting process was approved by NHSRC, protocol number (NHSRC) (#1099) in Malawi. This process was chosen because it protected participants from unwanted disclosure that would come due to paper copies of the consent form. Participants for IDIs and FGDs were compensated with the equivalent of 10USD for participation. All qualitative data were transcribed and translated into English for analysis.

### Analysis

MM listened to audios for rapid quality assessment and iterative feedback on IDI/FGD questions. Saturation was assessed through systematic coding.

We used a deductive approach, drawing on existing literature [1,2,23,24] to develop an initial codebook, and allowed inductive codes to emerge organically throughout the coding process. Four interviews and one FGD were coded in an iterative process and in consultation with authors, resulting in a final codebook. MM and RP applied the final codebook to the remaining interviews and FGDs. MM and RP summarized codes and identified overarching themes and used constant comparative methods to 1) compare themes by clients with or without IIT; and 2) compare themes by clients and HCWs to identify meaningful segments, patterns and variations and come up with final overarching themes.

## Results

### Quantitative analysis

**Fig 1:**
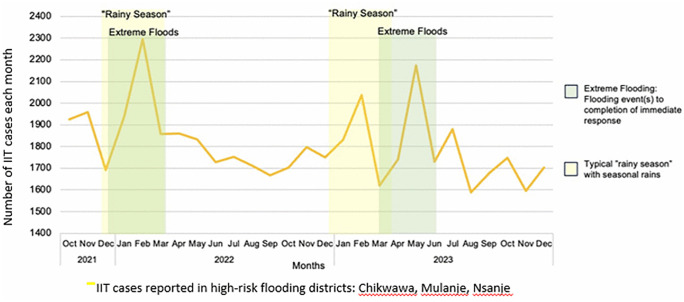
IIT cases in three districts in Malawi experiencing the highest flooding 2021-2023: Chikwawa, Mulanje and Nsanje.

Using routine program data from six high-risk flooding districts in Malawi, we examined IIT patterns over 27 months (October 2021 to December 2023), focusing on periods surrounding severe flooding events declared by the National Declaration of Flooding Emergency. A total of 49,152 IIT cases were observed across 27 months (Fig 1). The temporal relationship between flooding events and IIT spikes was consistent throughout the observation period, with distinct peaks coinciding with each major flooding episode.

During the first traditional rainy season and first extreme flooding period from late December 2021 through February 2022 (associated with Tropical Storm Ana), monthly IIT cases demonstrated a marked increase from pre-flooding IIT numbers: from an average of 1845 cases/month in October-December to a peak of 2300 cases/months in February 2022, the height of Tropical Storm Ana’s impact. This represents a 24% relative increase in IIT during community-wide flooding. Following the initial flooding event, IIT cases declined to a stabilized level of approximately 1675–1850 cases/month throughout mid- to late-2022.

During the second traditional rainy season from December 2022 to March 2023, there was a peak of 2045 IIT cases/month in February 2023, up from the average 1773 cases/month during non-rainy season months in 2022. This represents a 15% relative increase in IIT during the traditional rainy season. There was also a second extreme flooding period in 2023, from March through June (associated with Cyclone Gombe). Monthly IIT cases rose from approximately 1773 cases/month during the non-rainy season in 2023 to a peak of 2175 cases in May 2023, during the height of Cyclone Gombe’s impact, reflecting a 23% increase. Following the second flooding period, IIT cases declined and stabilized at approximately 1600–1750 cases/month for the remainder of 2023.

### Qualitative analysis

Between July and August 2024, we recruited 24 ART clients and conducted 23 IDIs with ART clients (12 with IIT, 11 matched without IIT) from six facility catchment areas. One client who did not experience IIT was not interviewed due to logistical challenges. We also conducted six FGDs with 34 HCWs from the same facilities. Among ART clients, 13 (57%) were female, the median age was 40 years (inter-quartile range [IQR] 28–46) and 21 (91%) were subsistence farmers. Nine clients (39%) experienced an IIT due to flooding and 17 (74%) were displaced from their homes, with the majority (n = 14, 82%) moving to government-supported camps. Among the HCWs, 17 (50%) were female and 18 (53%) were TS. HCWs were employed by MoH or implementing partners (including PIH) and had worked in the ART facility for a median of 18 (IQR 12–24) months.

Flooding presented multiple individual and health systems challenges to ART engagement.

#### Loss of property and income-generating activities.

Clients experienced major life disruptions due to flooding, losing homes, household belongings, and income-generation, including crops and livestock.

*“It was very hard for me to go to the facility and access my ART because I could not manage to cross the rivers. All seeds that I planted got washed away by floods, I also had goats and chickens which got washed away by floods as well.”- Female ART client who experienced IIT*

Flooding led to food insecurity, with the loss of maize stock (the staple crop) and maize fields. This increased pressure on some clients who believed they were unable to take ART without food.

*“ART medication needs food. It doesn’t work when you take ARVs without food. We struggled with life because we could leave the camp and go searching for food. Sometimes we could eat once in a day which [is] worrisome to ART medication”-Male ART client who experienced IIT*

As a result, clients depended heavily on donations from NGOs and emergency support from government. These resources did not meet basic needs, forcing clients to prioritize immediate survival needs over ART refills, travelling for work elsewhere.

*“Yes. I moved to Mozambique to earn a living. I could not come back to Malawi to get my treatment because I had no money for transport”- Male ART client who experienced IIT*

HCWs were not immune to the effects of flooding: over half lost homes and were forced to relocate to camps. Additionally, flooding led to increased workloads and exhaustion. Continuing to provide routine health services alongside flood-specific services placed additional stress on HCWs and their families, who did not receive aid because they were not viewed as vulnerable to the floods but as part of the relief effort.

*“We need support as health workers during this time, just like how the communities are supported. Like for instance, the previous floods, we were affected just like anyone and needed support to keep us going during this time.” – TS, Female*

Given the desperate situation, participants highlighted the need for a swift, comprehensive response, particularly for those living in remote rural areas.

*“An assessment should be done quickly to see the number of affected people and provide timely and necessary support. People from remote communities like us are abandoned. Support/aid for flood victims was focused on semi urban areas along the main roads leaving us with no hope and support” - Female ART client who experienced IIT*

#### Loss of ART medication and medical records.

Losing medication and medical records increased the risk of IIT. Most clients (n = 17, 77%) lost medication due to flooding, or left medication behind during evacuation, and over two-thirds lost medical records. Identifying individuals as ART clients and confirming their specific ART regimens was difficult, given the loss of paper records and non-functioning electronic medical records (EMR). Providing ART refills proved challenging due to reliance on phone calls to identify medication histories.

*“Flooding affected my ART badly because they got washed away. My health passport got washed away. I couldn’t remember my appointment date, because I used to ask children to check my date from the health passport. I had no peace of mind and I didn’t know what to do so I just stayed (at home)”- Female ART client who experienced IIT*

Flooding also caused frequent drug stock-outs. Given increased demand from clients who had lost their medication, HCWs had difficulty supplying medications at facilities and outreach clinics.

*“Sometimes it might happen that the area affected by floods has a lot of ART clients and most clients’ drugs have been washed away with floods, so that we used to have supply shortages. Because it happened that we took the drugs to the camps, we remained with very few stocks at the facility, meaning other clients will get affected as well” - ART provider, male*

Clients who did not experience IIT prepared for cases of flooding. They reported requesting more drug supply as well as keeping the medication in plastic bags and safe in case of floods.

*“When the heavy rains started, I had to take my ARVs which were wrapped in a plastic bag. This was because I thought that if I miss taking my ARVs for a single day, my viral load would be high. So I managed to keep my ARVs safe and move with them to the camp” – Female ART client who did not experience IIT.*

#### Limited physical access to health care.

Damaged roads, flooded rivers, and closed facilities made accessing care difficult, further increasing the risk of IIT.

*“For me to miss my appointment it was because of the floods that cut so many rivers and the waters were running so fast and it was very hard for one to cross so we could just turn back and stay. This is what caused me to stay that long without going to the facility.”- Female ART client who experienced IIT*

Health facilities were not spared the severe impacts of flooding, and those located within flood zones were closed for variable durations. One facility was filled with mud and debris, requiring clients to cross a flooded river to access care at an alternative facility.

*“… we stayed for a very long time without electricity at the facility and most of the services needed electricity; for instance, TB samples needed electricity and it was hard to keep those samples and transportation to the District Health Office could not be done right away. So, having these challenges, clients’ treatment was delayed.” - ART provider, male*

Despite this, some clients remained on treatment by travelling in dangerous conditions to get ART refills.

*“Yes. I could swim across the river to get my ART. It was risky but there was nothing else that I could do to get my ART; so skillfully, I managed to swim across the river” - Male ART client who did not experience IIT*

Others borrowed ART from friends and relatives.

*“Yes. I started feeling unwell and I was risking my life for not taking ART for days so I found someone who lent me some ART…, She gave me pills that I used while figuring out means to get my ART at the clinic… I saw the color and label of the bottle and the color of the pills”- Female ART client who did not experience IIT*

Some clients utilized guardians to collect ART from the facilities on their behalf.

*“I remember when I ran out of ARVs, my elder sister volunteered to go to the facility to get ARVs on my behalf despite that the rivers were flooded*.” *Female ART client who did not experience IIT*

To improve access to HIV care, some facilities established outreach clinics in government evacuation camps.

*“It was hard for clients to move from the camps to the facility to get their treatment. So we had to introduce outreach clinics to help clients who missed their appointment and those who lost their drugs due to flooding” - ART provider, male*

#### Lack of privacy of ART services.

The lack of privacy in overcrowded homes and camps during flooding presented major challenges for taking ART pills unobserved and increased the chance of IIT. Clients could also face unwanted disclosure when seeking care at outreach clinics.

*“It becomes a challenge for clients to take ARVs even if they have some because of being afraid of unwanted disclosure. In camps, people stay together in tents that makes it difficult for clients to swallow ARVs as a result they have days without taking ARVs due to lack of private space”- ART provider, female*

Despite this, some clients continued to take pills openly, accepting possible disclosure and stigma.

*“I did not feel shy. I could just take my bag, bring out ART and swallow without being worried of people. I did this because that is how I am and I could not hide it.” - Female ART client who did not experience IIT*

Other clients were motivated to stay healthy by taking ART. They continued to take ART although they had privacy concerns

*“I thought it wise not to be ashamed of myself whenever I wanted to swallow my ARVs because I realized that if I won’t be taking my ARVs, I may lose my life so I thought it was important for me to continue with my medication” -Male client who did not experience IIT” -Male ART client who did not experience IIT*

#### Absence of guidelines and facility policies on flood-preparedness.

HCWs highlighted the lack of national and local flood-preparedness guidelines, and the inadequacy of existing ART and facility operations guidelines. Due to limited staffing, HCWs struggled to meet patients’ needs in facilities and outreach clinics. They also encountered challenges in efficiently utilizing Ministry of Health vehicles to staff outreach clinics. In the absence of clear guidelines, HCWs reacted as best they could.

*“If we refer from 2022 ART guideline there is no topic called ART in emergency. So it is important for providers to be oriented on this. Yes, we do work to help clients but without following any guidelines we call it “phwanyaphwanya (willy-nilly)” - ART provider, male*

#### Lack of a coordinated and comprehensive response.

HCWs reported that agencies and NGOs delivering services for flood victims competed for shared facility resources such as vehicles and staffing. Within the camps, health service delivery frequently clashed with delivery of other services, sometimes resulting in clients needing to choose between two desperately-needed services.

*“You could see World Food Program distributing food at the camps and at the same time you are there to provide ART services so most clients would prefer to go and receive food donations first then later on their ART. It was very costly because sometimes you could go and help very few clients because of that yet you have used a lot of fuel, time and the like.” - ART provider, male*

The lack of coordination and leadership resulted in conflict between cadres of HCWs, and disorganized care delivery. TSs, responsible for tracing and client retention, felt excluded when NGOs dictated the composition of mobile clinic teams without coordination, undermining their ability to trace clients.

*“We need to have awareness where other NGOs would know or understand the roles and responsibilities of a TS, because they are not involved in outreach clinics yet we are the ones that are involved in counselling and tracing of clients...” – TS, male*

## Discussion

Using routine HIV program data, we found that the absolute number of IIT cases consistently increased during extreme flooding events, demonstrating the acute vulnerability of HIV treatment continuity to extreme weather events in these high-risk districts. In qualitative data, ART clients and HCWs in Malawi reported major life disruption due to flooding. IIT among ART clients increased by 35% during the flooding period and many clients were forced to relocate, largely to government camps. Clients and HCWs lost property and the ability to generate income through subsistence farming. Additional challenges were the loss of ART medication and medical records, drug stockouts, restricted access to facilities and limited privacy in outreach clinics and government camps. HCWs highlighted the absence of flood-preparedness guidelines, and the uncoordinated response. Despite these challenges, many clients managed to remain on ART. Clients and HCWs proposed solutions including the need for a rapid, coordinated response with national and local guidelines, particularly for remote, rural settings; stockpiling ART; six-month dispensing; access to a functioning EMR for ART client records; and provision of sufficient tents to ensure privacy during service delivery and allow clients to take ARVs with privacy.

Major disruptions of daily life from losses of crops, livestock and property forced most clients and many HCWs to relocate to camps. As almost all clients were subsistence farmers, flooding meant losing income-generating activities and relying entirely on mostly inadequate hand-outs from government or NGOs. Flooding exacerbated food insecurity, which has resulted in poorer health outcomes for PLHIV in other settings due to increased immune impairment [18,25]. Health system resilience during emergencies depends on HCWs’ safety and well-being [23,26]. However many HCWs were also directly impacted by the effects of flooding [12,27] but generally excluded from receiving aid as they were regarded as part of the emergency response. Participants called for a rapid, comprehensive response, particularly for those living in remote, rural areas, and including HCWs. Flood-preparedness policies should integrate food aid with ART delivery to flood survivors to reduce IIT.

In flooding, IIT is often caused by to limited physical access to health services [24,28,29]. In our study, restricted access to health facilities affected clients’ ability to receive ART refills, government’s capacity to replenish dwindling stocks, and HCWs’ ability to support clients [30]. Flood survivors had great difficulty in accessing medications because of local stockouts and limited physical access to health facilities, similar to circumstances in Mozambique during previous flooding disasters [31]. Loss of medication and health records during flooding events also increased the risk of IIT. In our study, three-quarters of clients lost ART, and two-thirds lost their medical records during floods and had to be contacted telephonically [24]. As has been suggested in other contexts [30,32], clients and HCWs advocated for a dual strategy to tackle the ART access obstacles: 1) multi-month dispensing (MMD) to provide six months of ART prior to the rainy season; and 2) stockpiling sufficient, appropriately stored reserves of ART to avoid supply chain disruptions. Providing MMD would decrease the burden on overstretched health facilities [32], and worked well in responding to the COVID-19 pandemic [23]. Additionally, HCWs felt that clients should be counselled on how to safely store their ART medication and health passports (personal health records).

Clear guidelines are urgently needed for an integrated response to flooding. The absence of national and local guidelines on flooding responses, and insufficient leadership in planning and resource allocation, hampered the health system’s ability to respond to the floods, similar to findings from Namibia [15]. The fragmented and uncoordinated response affected service delivery, as has been reported in numerous previous studies [30,33], and may have led to worse health outcomes. The lack of a national response is surprising given that Malawi is regularly subject to extreme weather events such as Tropical Storm Ana and cyclone Gombe, and was included as one of the most climate-vulnerable countries in the *Global Shield against Climate Risks* which was launched at the 2022 United Nations Climate Conference (COP27) [34].

In addition to guidelines, improved coordination between local and international NGOs in disaster settings is crucial [25,27,35]. One critical area for such coordination was the outreach clinic. In our study, as was the case in Namibia [23], outreach clinics were viewed as an acceptable option to access ART refills. However, while the Ministry of Health’s outreach clinics provided emergency general health services as well as HIV care and treatment, they lacked privacy. Different types of care and aid were offered within the same space, increasing the risk of unwanted disclosure of HIV status, and possibly inhibiting some clients from collecting ARV medication. Coordinating ART services in outreach clinics with delivery of other aid might prevent clients having to prioritize one much-needed service over another. To address privacy issues, a comprehensive flood-preparedness response should include the timely provision of spaces for private service provision. Dispensing ART in unmarked packets could also prevent unwanted disclosure [32].

Our findings represent the opinions of critical stakeholders living in flood-prone districts and may inform the development of a proactive, effective response to the impact of flooding on ART services in Malawi and other vulnerable, high HIV prevalence settings. Our study was strengthened by the inclusion of the lived experiences of health care workers as well as ART clients, offering a timely and comprehensive perspective on access to HIV care during flooding. Adding to the limited literature on such lived experiences, the study offers a timely and comprehensive perspective on access to care during community-wide flooding.

Our study had certain limitations. Although we used mixed methods, we only used quantitative data to describe the patterns of IIT. No additional quantitative data were collected to explore other factors that may impact on flooding and access to care. The study used purposive sampling for IDIs and FGDs and therefore the responses may have been affected by social desirability bias. Finally, the study was conducted in three districts in one of the three regions in Malawi, and respondents’ experiences may not represent all people affected by flooding across the country and elsewhere.

Our study reflects the devastating impact of flooding on the precarious lives of ART clients and HCWs in Malawi, who lost property and were often unable to generate their usual income. Forced migration to government camps further disrupted their lives, while destruction of infrastructure and transport hazards prevented access to ART services, resulting in IIT and increasing the risk of severe illness. Developing resilient healthcare systems requires supporting HCWs, who were also severely impacted by such emergencies and received insufficient aid. Urgent solutions are required for populations that are particularly vulnerable to the impact of flooding. Such solutions may include MMD, pre-emptive stockpiling of medications prior to flooding periods, and the formulation of a comprehensive response with national and local guidelines. Outreach clinics should be tailored to personal and community health needs.

## Supporting information

S1 File Codebook. (XLSX)
